# PD-1抑制剂治疗复发或难治性经典型霍奇金淋巴瘤的疗效和安全性分析

**DOI:** 10.3760/cma.j.issn.0253-2727.2023.07.005

**Published:** 2023-07

**Authors:** 丹丹 山, 慧敏 刘, 薇 刘, 文阳 黄, 瑞 吕, 书会 邓, 树华 易, 刚 安, 燕 徐, 伟薇 隋, 婷玉 王, 明伟 傅, 耀中 赵, 录贵 邱, 德慧 邹

**Affiliations:** 1 中国医学科学院血液病医院（中国医学科学院血液学研究所），实验血液学国家重点实验室，国家血液系统疾病临床医学研究中心，细胞生态海河实验室，天津 300020 State Key Laboratory of Experimental Hematology, National Clinical Research Center for Blood Diseases, Haihe Laboratory of Cell Ecosystem, Institute of Hematology & Blood Diseases Hospital, Chinese Academy of Medical Sciences & Peking Union Medical College, Tianjin 300020, China; 2 天津医学健康研究院，天津 301600 Tianjin Institutes of Health Science，Tianjin 301600, China

**Keywords:** 程序性细胞死亡蛋白-1抑制剂, 复发或难治, 经典型霍奇金淋巴瘤, PD-1 inhibitor, relapsed/refractory, Hodgkin's lymphoma

## Abstract

**目的:**

回顾性分析程序性细胞死亡蛋白-1（programmed cell death protein-1，PD-1）抑制剂单药或联合化疗治疗复发或难治性经典型霍奇金淋巴瘤（R/R cHL）的疗效和安全性。

**方法:**

共纳入2016年9月至2020年12月就诊于中国医学科学院血液病医院的35例R/R cHL患者，其中17例给予PD-1抑制剂单药治疗，另18例接受PD-1抑制剂联合化疗。回顾性分析其临床资料和随访数据，采用Kaplan-Meier法和Cox比例风险模型进行生存分析。

**结果:**

35例R/R cHL患者中位年龄29（11～61）岁，男性占54.3％，62.9％的患者Ann Arbor分期为进展期，48.6％伴有结外侵犯。PD-1抑制剂治疗前的中位治疗线数为2（1～3）线。28例患者获得客观缓解［其中22例为完全缓解（CR）］，客观缓解率（ORR）和CR率分别为80.0％和62.9％；其中PD-1单药治疗组的ORR和CR率分别为64.7％和58.8％，PD-1联合化疗组的ORR和CR率分别为94.4％和66.7％。18例［13例CR和5例部分缓解（PR）］患者序贯自体造血干细胞移植（auto-HSCT）治疗，其中8例患者auto-HSCT后给予PD-1抑制剂单药巩固治疗；移植后患者均获得并维持CR状态，与未序贯auto-HSCT的患者相比无进展生存（PFS）率显著升高（4年PFS率分别为100％和53.5％，*P*＝0.041）。免疫相关的不良事件发生率为29％，仅1例患者出现≥3级不良反应，整体安全性良好。

**结论:**

PD-1抑制剂治疗R/R cHL安全、有效，PD-1抑制剂联合化疗显著提高缓解率。对于挽救治疗敏感的患者，auto-HSCT巩固治疗进一步改善长期生存。

## Abstract (en)

**Objective:**

This retrospective, single-center study aimed to evaluate the efficacy and safety of programmed death-1（PD-1）inhibitors, either as monotherapy or in combination with chemotherapy, in the management of relapse/refractory classical Hodgkin's lymphoma（R/R cHL）.

**Methods:**

A total of 35 patients with R/R cHL who received treatment at the Institute of Hematology and Blood Diseases Hospital, Chinese Academy of Medical Sciences & Peking Union Medical College from September 2016 to December 2020 were enrolled in this study. Among them, 17 patients received PD-1 inhibitor monotherapy（PD-1 inhibitor group）, while 18 patients received a combination of PD-1 inhibitor and chemotherapy（PD-1 inhibitor + chemotherapy group）. Clinical data and follow-up information were retrospectively analyzed, and survival analysis was conducted using the Kaplan-Meier method and Cox proportional hazards model.

**Results:**

The median age of the 35 patients with R/R cHL was 29 years（range: 11–61 years）, with 54.3％ being male. According to the Ann Arbor staging system, 62.9％ of patients presented with advanced（stage Ⅲ/Ⅳ）disease, and 48.6％ had extranodal involvement. Before PD-1 inhibitor therapy, the median number of prior lines of therapy was 2（range: 1–3）. Objective responses were observed in 28 patients, including 22 complete response（CR）cases, resulting in an overall response rate（ORR）of 80.0％ and a CR rate of 62.9％. Specifically, the ORR and CR rates were 64.7％ and 58.8％, respectively, in the PD-1 inhibitor group and 94.4％ and 66.7％, respectively, in the PD-1 inhibitor + chemotherapy group. Among the 18 patients who underwent sequential autologous hematopoietic stem cell transplantation（auto-HSCT）［13 CR and five partial response（PR）cases］, eight patients received PD-1 inhibitor therapy after auto-HSCT as consolidation therapy. All patients maintained a CR status after transplantation, and they exhibited significantly improved progression-free survival（PFS）rates compared with those who did not undergo sequential auto-HSCT（4-year PFS rates: 100％ *vs* 53.5％; *P*＝0.041）. The incidence of immune-related adverse events was 29％, with only one patient experiencing grade≥3 adverse reactions, which indicated a favorable safety profile for the treatment approach.

**Conclusion:**

PD-1 inhibitor monotherapy demonstrates notable efficacy and sustained response in patients with R/R cHL. PD-1 inhibitors combined with chemotherapy significantly improve response rates. Additionally, for salvage therapy-sensitive patients, consolidation treatment with PD-1 inhibitors after auto-HSCT exhibits the potential for prolonging PFS.

经典型霍奇金淋巴瘤（cHL）是一种治愈率较高的淋巴系统肿瘤，化疗和放疗是目前主要的治疗手段。5％～10％的cHL患者在初始诱导治疗后无法获得缓解，10％～30％的患者获得缓解后发生疾病复发/进展。大剂量化疗联合自体造血干细胞移植（auto-HSCT）仍然是化疗敏感的年轻复发或难治性（R/R）cHL患者的标准挽救治疗方案，但只有约半数患者能够获得持久的疾病控制[1]；auto-HSCT前缓解质量是最相关的预后因素之一，获得完全代谢缓解的患者疗效更佳[2]。在霍奇金淋巴瘤Reed-Sternberg（RS）细胞中，9p24.1染色体的扩增和Epstein-Barr（EB）病毒感染导致PD-1的配体PD-L1/PD-L2过度表达，与T细胞上的PD-1受体结合，进而抑制T细胞的激活和增殖，最终导致T细胞衰竭，诱导免疫逃逸[2]。PD-1或PD-L1抑制剂可阻断PD-1/PD-L1通路，逆转免疫逃逸，从而发挥抗肿瘤作用。国内自主研发的PD-1抑制剂如信迪利单抗、替雷利珠单抗和卡瑞利珠单抗等均先后被国家药品监督管理局批准用于治疗至少经过二线系统化疗的R/R cHL患者，但国内目前尚缺乏较大系列的临床应用报告。本研究旨在评估单中心PD-1抑制剂治疗R/R cHL的疗效和安全性，探索PD-1抑制剂桥接auto-HSCT及移植后PD-1抑制剂巩固治疗的疗效和安全性。

## 病例与方法

1. 病例：回顾性分析2016年9月至2020年12月就诊于我中心的35例R/R cHL患者，所有患者均经病理活检明确诊断，诊断依据参照《中国霍奇金淋巴瘤的诊断与治疗指南（2022年版）》[3]，并且经过PET-CT、CT、骨髓穿刺等检查明确分期及国际预后评分（IPS）。

2. 治疗方案：患者之前未接受过免疫检查点抑制剂（包括CTLA-4、PD-1/PD-L1抑制剂）治疗。挽救治疗给予PD-1抑制剂单药或联合化疗，联合化疗包括以吉西他滨为基础的方案［如GemOx方案（吉西他滨、奥沙利铂）、GDPE方案（吉西他滨、地塞米松、顺铂、依托泊苷）和IGEV方案（异环磷酰胺、吉西他滨、长春瑞滨、泼尼松）］或低剂量地西他滨（DP方案）。不同PD-1抑制剂的具体用法为：信迪利单抗200 mg，每3周1次；替雷利珠单抗200 mg，每3周1次；卡瑞利珠单抗200 mg，每2周1次。

对于挽救化疗敏感、适合移植且先前未接受过移植治疗的患者，推荐auto-HSCT巩固治疗；同时对于具有高危复发因素的患者，推荐auto-HSCT后予PD-1抑制剂巩固治疗，启动时间为至少移植后60 d或auto-HSCT相关不良反应恢复后。具有高危复发因素定义为符合以下≥1个危险因素：①原发难治性cHL［定义为一线标准治疗未能达到完全缓解（CR）］；②一线治疗后1年内复发；③复发时伴结外受累；④复发时出现B症状；⑤需要>1线挽救治疗；⑥auto-HSCT前未达到CR。

对于不适合移植或无移植意愿的患者，PD-1抑制剂单药或DP方案持续治疗至2年或复发/进展或不良反应不能耐受，联合化疗者接受4～6个周期诱导化疗随后序贯PD-1抑制剂单药持续治疗至2年或复发/进展或不良反应不能耐受。

3. 疗效与安全性评估：疗效评价依据2014年Lugano疗效评价标准[4]，于基线和治疗后行PET-CT检查，依据Deauville评分系统进行疗效评价，分为CR、部分缓解（PR）、疾病稳定（SD）、疾病进展（PD），以CR+PR为有效。药物不良反应参考美国国立癌症研究所CTCAE 5.0版和《美国国立综合癌症网络免疫治疗相关毒性的管理指南2021版》进行评价。

4. 生存随访：通过查阅门诊、住院病历及电话联系进行随访。随访截止日期为2021年12月。无进展生存（PFS）时间定义为自接受PD-1抑制剂治疗至疾病进展或死亡或末次随访的间隔时间。总生存（OS）时间定义为从接受PD-1抑制剂治疗起至任何原因导致患者死亡或末次随访的间隔时间。

5. 统计学处理：应用IBM SPSS Statistics 23.0软件进行统计分析。采用Kaplan-Meier法绘制生存曲线，并进行Log-rank检验，检验水准：*α*＝0.05，*P*<0.05为差异有统计学意义。

## 结果

1. 患者基线特征：共纳入了2016年9月至2020年12月就诊于我院的35例R/R cHL患者。患者中位年龄29（11～61）岁，男19例（54.3％），女16例（45.7％），62.9％的患者Ann Arbor分期为进展期，48.6％伴有结外侵犯，97.1％的患者有高危复发因素。主要病理学类型为结节硬化型（74.2％）和混合细胞型（22.6％）。PD-1抑制剂治疗前的中位治疗线数为2（1～3）线，中位化疗周期数为7（2～16）个，主要治疗方案包括ABVD（80.0％）、BEACOPP（8.6％）、IGEV（5.7％），所有患者之前未接受过PD-1抑制剂治疗。

2. 治疗与治疗反应及生存：随访截至2021月12月，中位随访时间为34（12～63）个月，无失访病例。28例患者获得治疗反应（CR+PR，其中22例为CR），ORR和CR率分别为80.0％（95％ *CI* 66.1％～93.9％）和62.9％（95％ *CI* 46.0％～79.7％）。中位PFS时间和OS时间均未达到；2例患者死亡，死亡原因分别为疾病进展和肺部感染。1年和预期3年PFS率均为79.7％；1年和预期3年OS率分别为97.1％和93.8％（图1）。

**图1:**
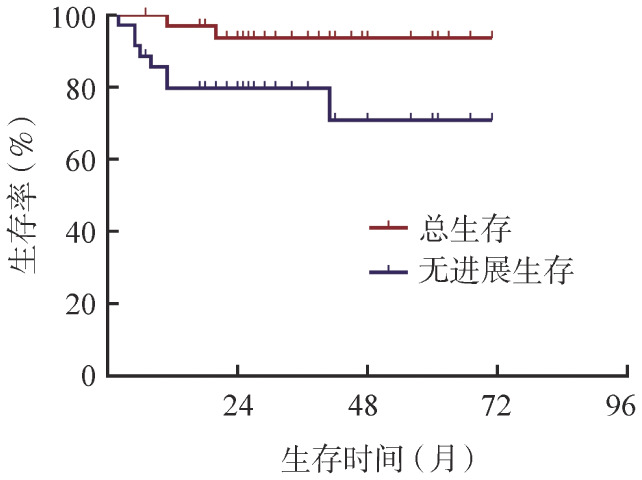
35例复发或难治性经典型霍奇金淋巴瘤患者的总生存和无进展生存曲线

PD-1抑制剂单药组共17例，接受PD-1抑制剂前中位治疗线数为2（1～3）线，中位PD-1抑制剂治疗周期数为18（1～97）个。ORR和CR率分别为64.7％（95％ *CI* 39.4％～90.0％）和58.8％（95％ *CI* 32.7％～84.9％）。中位随访45个月，1年、3年和4年预期PFS率分别为70.6％、70.6％和60.5％，1年、3年和4年预期OS率均为94.1％；仅1例（5.9％）患者因疾病进展死亡。PD-1抑制剂联合化疗组共18例，ORR和CR率分别94.4％（95％ *CI* 82.7％～100.0％）和66.7％（95％ *CI* 42.5％～90.8％）；联合治疗组的ORR显著高于单药组（*P*＝0.041），CR率差异无统计学意义（*P*＝0.733）。联合治疗组的预计4年PFS率为88.5％，与单药组比较差异无统计学意义（*P*＝0.165）。其中联合以吉西他滨为基础的化疗共13例（GemOx方案6例，GDPE方案6例，IGEV方案1例），接受PD-1抑制剂前中位治疗线数为2（2～3）线，中位治疗周期数为3（2～8）个；中位随访25个月，ORR为92.3％（95％ *CI* 75.5％～100.0％），CR率为53.8％（95％ *CI* 22.5％～85.2％），1年和预期3年PFS率均为88.5％。DP方案组5例，中位治疗线数为3（2～4）线，中位治疗周期数为5（3～19）个；中位随访17个月，所有患者均获CR，预期2年PFS率为100％。

28例获得治疗反应的患者，其中18例后续序贯auto-HSCT，均为具有高危复发因素的患者，其中≥2个高危因素者13例。移植前获得CR和PR的患者分别为13和5例，移植后中位随访37个月，所有患者均获得并维持CR状态；其中8例患者（≥2个高危因素者7例）auto-HSCT后给予PD-1抑制剂单药巩固治疗（6例信迪利单抗，2例替雷利珠单抗），中位巩固周期数为5.5（1～16）个，至随访结束所有患者均为持续CR。与未序贯auto-HSCT的患者相比，移植组患者PFS率显著升高（4年PFS率：100％对53.5％；*P*＝0.041）（图2）。

**图2:**
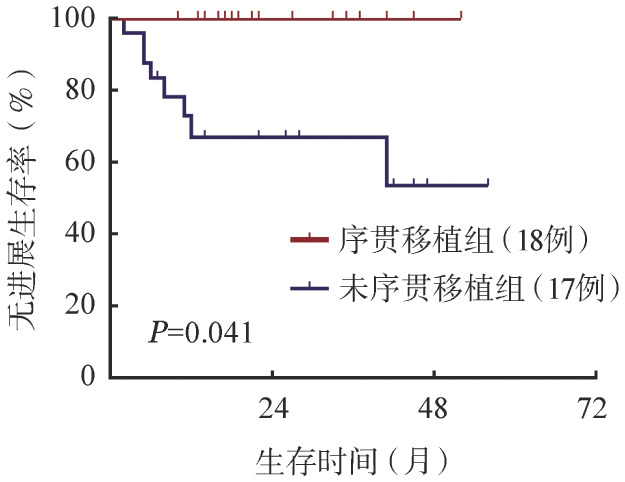
PD-1抑制剂序贯移植对复发或难治性经典型霍奇金淋巴瘤患者无进展生存的影响

3. 免疫相关的不良事件（irAE）：29％的患者出现了irAE。在PD-1抑制剂单药治疗中，irAE的发生率为35％，包括反应性皮肤毛细血管增生症（1级，18％，均发生于卡瑞利珠单抗治疗者）、血淀粉酶升高（2级，6％）、血脂肪酶升高（3级，6％）、甲状腺功能减退（1级，6％）、总胆红素升高（2级，6％）和瘙痒（1级，6％）。在PD-1抑制剂联合化疗组，irAE的发生率为22％，包括肺炎（2级，6％）、血淀粉酶升高（2级，6％）、皮疹（1级，6％）、肝功能异常（1级，6％）和总胆红素升高（1级，6％）。1例患者出现3级不良反应（血脂肪酶升高，3％），未经特殊治疗恢复正常，没有因不良反应终止或延迟治疗者。接受auto-HSCT的患者无一例发生明显的植入综合征。

## 讨论

对于诱导治疗失败的年轻、适合移植的cHL患者，auto-HSCT仍然是标准的挽救治疗策略，约50％的患者有效；auto-HSCT前缓解质量是最相关的预后因素之一[5]–[6]。联合新药如维布妥昔单抗（Brentuximab Vedotin, BV）和PD-1抑制剂等，能够提高挽救治疗的有效率和CR率，使更多的患者有机会序贯auto-HSCT治疗并同时提高了整体疗效[5]–[6]。

纳武利尤单抗（Nivolumab，Nivo）和帕博利珠单抗（Pembrolizumab，Pembro）是已获美国食品药品监督管理局批准用于治疗R/R cHL的PD-1抑制剂，单药治疗至少经过二线系统化疗或auto-HSCT后的R/R cHL患者的ORR分别为69％和71.9％，但CR率较低，分别为16％和27.6％[5]–[6]。信迪利单抗、替雷利珠单抗和卡瑞利珠单抗均是国内自主研发的PD-1抑制剂，临床试验结果显示单药治疗类似的R/R cHL患者的ORR分别为80.4％、87.1％和76.0％，CR率分别为34％、67.1％和26.7％[7]–[9]，均先后被国家药品监督管理局批准用于治疗至少经过二线系统化疗的R/R cHL患者。本研究中17例患者使用国产PD-1抑制剂单药治疗，之前中位治疗线数为2（1～3）线，ORR和CR率分别为64.7％和58.8％，疗效与临床试验结果相当。

PD-1抑制剂单药使用存在CR率偏低的问题，且大部分患者终将复发；Nivo和Pembro单药治疗的5年PFS率均<20％，替雷利珠单抗和卡瑞利珠单抗治疗获得CR或治疗反应者3年PFS率分别为52.1％和54.4％[5]–[6],[8]–[9]。传统二线化疗方案，包括以铂类或吉西他滨为基础的方案，治疗R/R cHL患者的ORR为67％～89％，CR率为17％～73％；而加入新药（如BV或PD-1抑制剂）或新药的联合方案（如BV联合PD-1抑制剂），明显提高R/R cHL患者挽救治疗的ORR（82％～100％）和CR率（61％～100％）[10]–[15]；同时有更高比例的患者有机会序贯auto-HSCT巩固治疗。Moskowitz等[10]应用Pembro联合GVD（吉西他滨、长春瑞滨、脂质体阿霉素）方案二线治疗39例R/R cHL患者，原发难治和1年内早期复发的患者比例分别为49％和38％。ORR和CR率分别高达100％和95％，其中92％的患者2个周期后即获得CR。95％的患者序贯auto-HSCT巩固治疗，移植后中位随访12.5个月，均持续CR。国内Wang等[16]–[17]对比分析低剂量去甲基化药物地西他滨联合卡瑞利珠单抗（DP）和卡瑞利珠单抗单药治疗≥2线治疗后R/R cHL患者的疗效。对于之前未接受过PD-1抑制剂的患者，DP方案的CR率显著高于单药组（79％对32％，*P*＝0.001）；中位随访34.5个月，DP组的PFS期也显著优于单药组（35个月对15.5个月，*P*＝0.020）。由于新药挽救治疗取得的巨大成功，对于获得CR的患者auto-HSCT序贯巩固治疗的作用尚不明确。目前尚缺乏BV/PD-1抑制剂联合化疗后未接受序贯auto-HSCT巩固化疗患者的长期随访结果。Mei等[18]进行的Nivo联合ICE方案（异环磷酰胺、卡铂、依托泊苷）的研究中，拒绝行auto-HSCT的4例患者中3例复发；Advani等[19]的BV联合Nivo的研究中仅有5例患者未行auto-HSCT巩固治疗，其中2例在治疗中已进展，另外2例患者最佳治疗反应仅PR。我们的研究中，18例接受PD-1抑制剂联合化疗患者的ORR显著高于17例接受PD-1抑制剂单药治疗的患者；18例伴有高危复发危险因素的患者（包括5例移植前PR的患者），移植后均获得CR并持续维持CR状态，较未行auto-HSCT患者PFS率显著提高（4年PFS率：100％对53.5％，*P*＝0.041）。提示对于R/R cHL患者，PD-1抑制剂联合化疗显著提高缓解率；对于挽救治疗敏感，尤其伴有高危复发危险因素的患者，auto-HSCT巩固治疗进一步延长和改善长期生存。但因本研究病例数较少，该结论仍需在更大规模人群中验证。

有研究显示auto-HSCT后的巩固治疗延长伴有高危复发因素患者的生存。AETHERA研究[20]显示合并高危复发因素的R/R cHL患者，BV巩固治疗组较安慰剂组PFS时间显著延长（42.9个月对24.1个月，*HR*＝0.57）。一项Ⅱ期临床研究中[21]，30例合并高危复发因素的R/R cHL患者auto-HSCT后接受8个周期Pembro巩固治疗，18个月的PFS率为82％，高于历史对照；本研究中8例患者（其中≥2个高危因素者7例）auto-HSCT后给予PD-1抑制剂单药巩固治疗，中位巩固周期数为5.5（1～16）个，至随访结束，所有患者均为持续CR，但尚缺乏随机、对照研究结果。

PD-1抑制剂临床应用中，除了常见的药物不良反应外，需特别关注免疫检查点抑制剂相关的毒性。本研究中irAE的发生率为29％，3级以上不良反应发生率为3％。所有的不良反应均能得到控制，没有患者因不良反应而中断治疗，提示整体安全性和耐受性良好。

综上所述，本研究结果显示PD-1抑制剂治疗R/R cHL安全、有效，PD-1抑制剂联合化疗显著提高缓解率。对于挽救治疗敏感的患者，auto-HSCT巩固治疗进一步延长和改善长期生存。
